# Visualizing drug-induced Stevens-Johnson syndrome: a clinical image

**DOI:** 10.11604/pamj.2024.48.183.44544

**Published:** 2024-08-19

**Authors:** Shivali Kalode, Prerna Tekulwar

**Affiliations:** 1 Department of Pathology, Jawaharlal Nehru Medical College, Datta Meghe Institute of Higher Education and Research, Sawangi (Meghe), Wardha, Maharashtra, India

**Keywords:** Steven-Johnson syndrome, drug-induced, gentamycin, purpuric macules

## Image in medicine

Steven Johnson syndrome (SJS) is an acute, self-limited condition that manifests as severe mucosal erosions with cutaneous macules that are erythematous and spread widely. Although being uncommon with an annual incidence of 0.05 to 2 cases per million people SJS has a significant impact on public health due to its high rates of morbidity and mortality. Here, we are presenting a case of a 53-year-old male, who presented in the dermatology department with a chief complaint of fever and extensive rashes over the trunk and bilateral upper limbs for a day. It was also associated with pain which was sudden in onset, burning type, continuous, localized, and severe in intensity, aggravated on touching with no relieving factor. The past history of the patient revealed that he took an injection of gentamycin intramuscularly from a general practitioner and developed this type of reaction. The patient was well-oriented and on examination, had hyperpyrexia, and generalized cutaneous eruptions on the trunk and bilateral upper limbs. This cutaneous eruption consists of purpuric macules which were tender on touch. All routine investigations were done and were in normal limits except for the raised erythrocyte sedimentation rate (ESR). Further, the skin biopsy was performed and sent to the histopathology section in the department of pathology. The labeled skin biopsy consists of a single, irregular, brownish tissue piece with skin attached measuring 0.5 x 0.5 cm. Microscopically, hematoxylin and eosin (H&E) staining revealed predominantly full-thickness necrosis and necrotic keratinocytes with few red blood cells, pigment incontinence, and parakeratosis, features suggestive of drug-induced Stevens-Johnson syndrome. The patient was treated under the expert guidance of a dermatologist with systemic steroids, Inj. Prednisolone 10 mg *qid* (four times a day) for 7 days was gradually tapered to 10 mg *tid* (three times a day) for 7 days, 10 mg *bid* (twice a day) for 5 days, then Tab Prednisolone 10 mg once daily for 5 days respectively.

**Figure 1:**
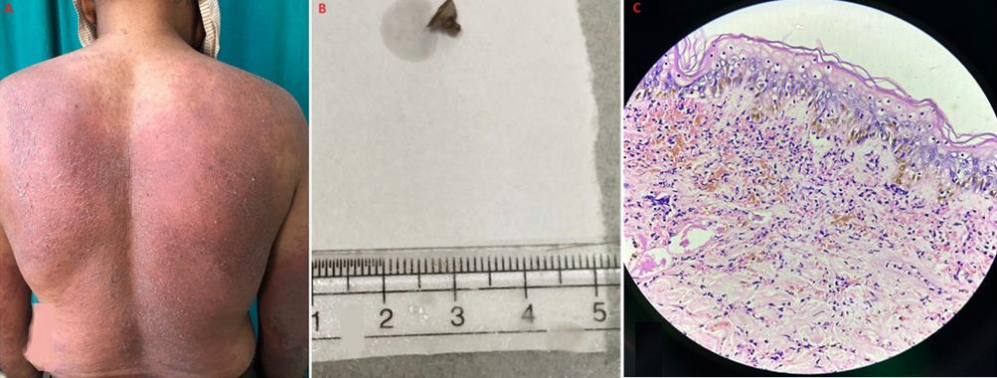
A) cutaneous eruptions consisting of purpuric macules; B) the skin biopsy; C) microscopic image at high power view (40X), H&E staining revealed predominantly full-thickness necrosis and necrotic keratinocytes with few red blood cells, pigment incontinence, and parakeratosis

